# Polymorphic sites preferentially avoid co-evolving residues in MHC class I proteins

**DOI:** 10.1371/journal.pcbi.1006188

**Published:** 2018-05-21

**Authors:** Linda Dib, Nicolas Salamin, David Gfeller

**Affiliations:** 1 Department of Oncology, Ludwig Institute for Cancer Research, University of Lausanne, Switzerland; 2 Swiss Institutes of Bioinformatics, Quartier Sorge, Lausanne, Switzerland; 3 Department of Computational Biology, University of Lausanne, Lausanne, Switzerland; University College London, UNITED KINGDOM

## Abstract

Major histocompatibility complex class I (MHC-I) molecules are critical to adaptive immune defence mechanisms in vertebrate species and are encoded by highly polymorphic genes. Polymorphic sites are located close to the ligand-binding groove and entail MHC-I alleles with distinct binding specificities. Some efforts have been made to investigate the relationship between polymorphism and protein stability. However, less is known about the relationship between polymorphism and MHC-I co-evolutionary constraints. Using Direct Coupling Analysis (DCA) we found that co-evolution analysis accurately pinpoints structural contacts, although the protein family is restricted to vertebrates and comprises less than five hundred species, and that the co-evolutionary signal is mainly driven by inter-species changes, and not intra-species polymorphism. Moreover, we show that polymorphic sites in human preferentially avoid co-evolving residues, as well as residues involved in protein stability. These results suggest that sites displaying high polymorphism may have been selected during vertebrates’ evolution to avoid co-evolutionary constraints and thereby maximize their mutability.

## Author summary

Amino acid co-evolution represents cases of simultaneous substitution of amino acids at distinct positions in protein sequences. In the MHC-I protein family, such co-evolution could result from either amino acid changes across species or changes within species due to the high polymorphism of MHC-I molecules. Here we show that signals captured by global methods such as Direct Coupling Analysis (DCA) to estimate co-evolution primarily result from changes across species. Moreover, our results indicate that polymorphic sites in MHC-I molecules tend to be decoupled from co-evolving ones. This could suggest that they have been selected to maximize their mutability, which is known to be functionally important to entail MHC-I molecules with a wide repertoire of binding specificities for antigen presentation.

## Introduction

Major Histocompatibility Complex class I proteins (MHC-I), also referred to as Human Leukocyte Antigen class I (HLA-I) in human, are expressed on the surface of cells. MHC-I proteins form a complex with either ‘self’ ligands derived from the endogenous proteins or ‘foreign’ ligands (non-self) derived from invading pathogens or somatic alterations in cancer cells. Upon presentation of non-self ligands from inside the cytoplasm, the complex can be recognized by CD8 T-cells [1]. MHC-I proteins show a very high degree of polymorphism especially around the peptide-binding groove and tens of thousands of different alleles are reported in databases like PFAM [2] or IMGT/HLA [3]. Moreover, striking differences in binding specificity are observed between different alleles. Several evolutionary events contributed to MHC-I diversity in vertebrates. Duplication events occurred during the evolution of jawed vertebrate, which led to MHC-I polygenicity in many species [4,5]. Following the gene duplication events, the different gene copies diverged through separate evolutionary processes, which allowed some MHC-I genes to gain different functions, while others became dysfunctional or lost [6]. Consequently, the number of MHC-I loci differs between vertebrate species [7]. These duplication events produced 6 MHC-I genes in human all located on chromosome 6. Three of them (HLA-A, HLA-B and HLA-C) are broadly expressed in most cell types and are the main contributors to class I antigen presentation. The high level of allelic diversity of the MHC-I in vertebrate population is likely due to strong selection because of the exposure of vertebrate populations to various infections across the world [8] [9]. In particular, the polygenicity and polymorphism entails the immune system of each individual with the ability to present at the cell surface a wide range of peptides from foreign pathogens.

Despite their high polymorphism, MHC-I alleles share the same three-dimensional fold across vertebrates. In particular, the peptide-binding groove is composed of two almost parallel alpha helices and one beta sheet. This conserved structure across all MHC-I alleles suggests that they undergo molecular constraints. Molecular constraints can be predicted using stability models that investigate the impact of a mutation on the structure (e.g. alanine scanning) [10] or conservation [11]. Recent studies have also demonstrated that simultaneously evolving sites (also called co-evolving sites) can reveal structural contacts [12] folding intermediate [13], allosteric communication, core protein sites [14], or functionally important sites [15]. Several models are available in the literature to predict co-evolving sites. Most of the models evaluate a score to assess if a pair of sites simultaneously evolves regardless of the other residues. Some of these models use statistical formalisms such as Mutual Information [16], Statistical Coupling Analysis [17] or Coev [14,18] when others use combinatorial formalism [19,20]. The only model that investigates co-evolving residues in the light of global alignment is Direct Coupling Analysis (DCA) [12], also introduced in the EVfold suite [21]. This phylogeny-free method was shown to accurately identify sites in contact in protein structures, and because of this, DCA has been used to help predicting protein structures [21][22][23][24].

In this work, we study the co-evolving constraints on MHC-I across vertebrates’ species using DCA. Despite the low number of species (<500), we observed that DCA could accurately predict structural contacts directly from MHC-I protein sequence alignment. We then investigated the relationship between polymorphism and co-evolution constraints. Our work reveals that polymorphism within human does not contribute much to the observed co-evolution signal. Moreover polymorphic sites show little overlap with both co-evolving sites across vertebrates and sites predicted to be most important in protein structural stability. We further extended the DCA algorithmic framework to incorporate multiple MHC-I ligands per allele and observed the same uncoupling between co-evolving and polymorphic residues. These results suggest that polymorphic residues in MHC-I molecules preferentially avoid sites displaying strong stability or co-evolutionary constraints.

## Results

### Co-evolution among MHC-I residues

**Fig 1:**
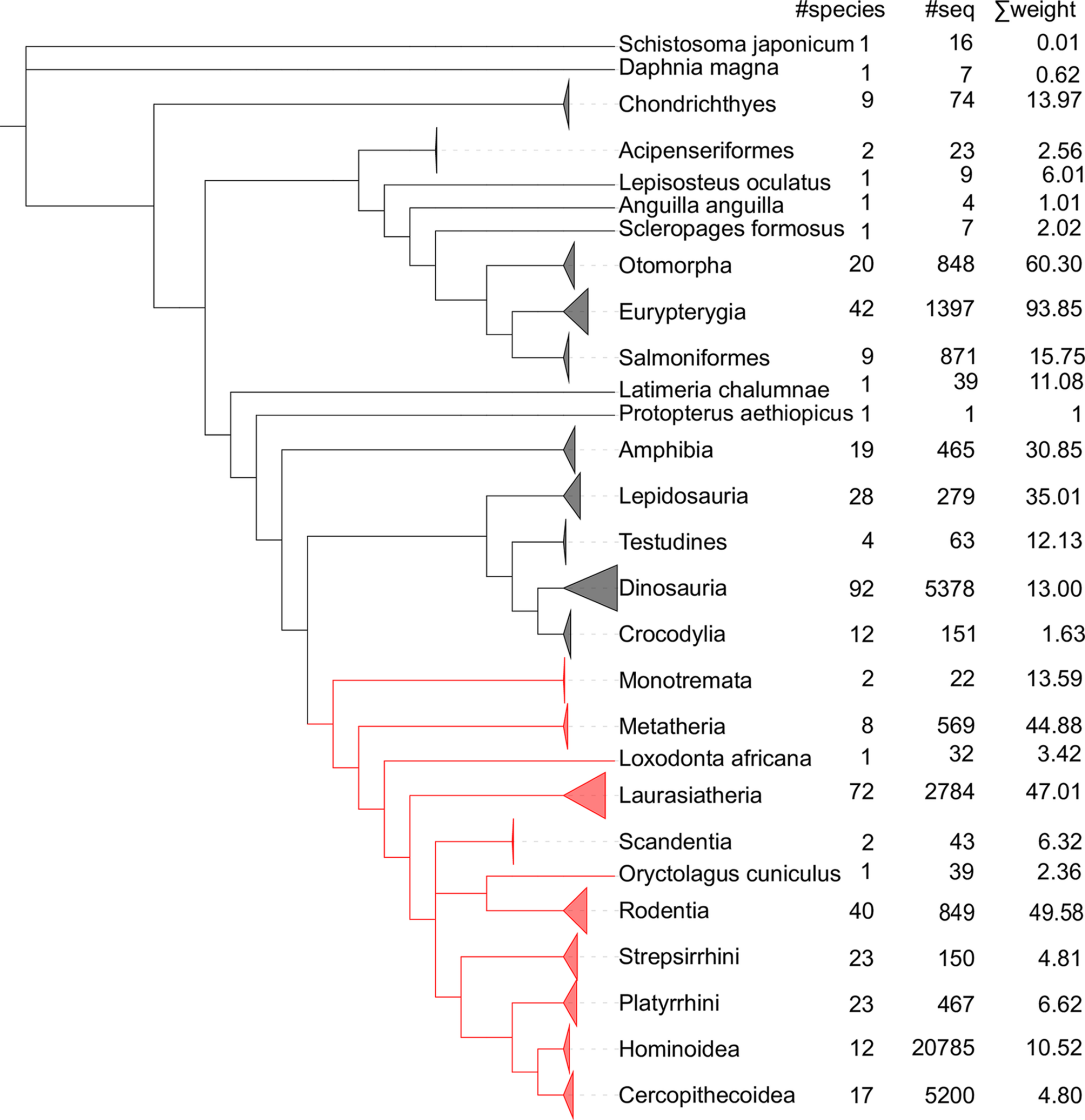
Species tree with number of sequences. Topological species tree issued from phyloT that illustrates the 445 vertebrate species represented in PFAM MHC-I alpha 1 and 2 domain family (PF00129). The number of sequences (column 2) and the number of species (column 1) per clade are indicated on the right. In red, we highlighted the mammalian clades. The sum of the weights in DCA of all sequences in each clade is shown in the last column.

**Fig 2:**
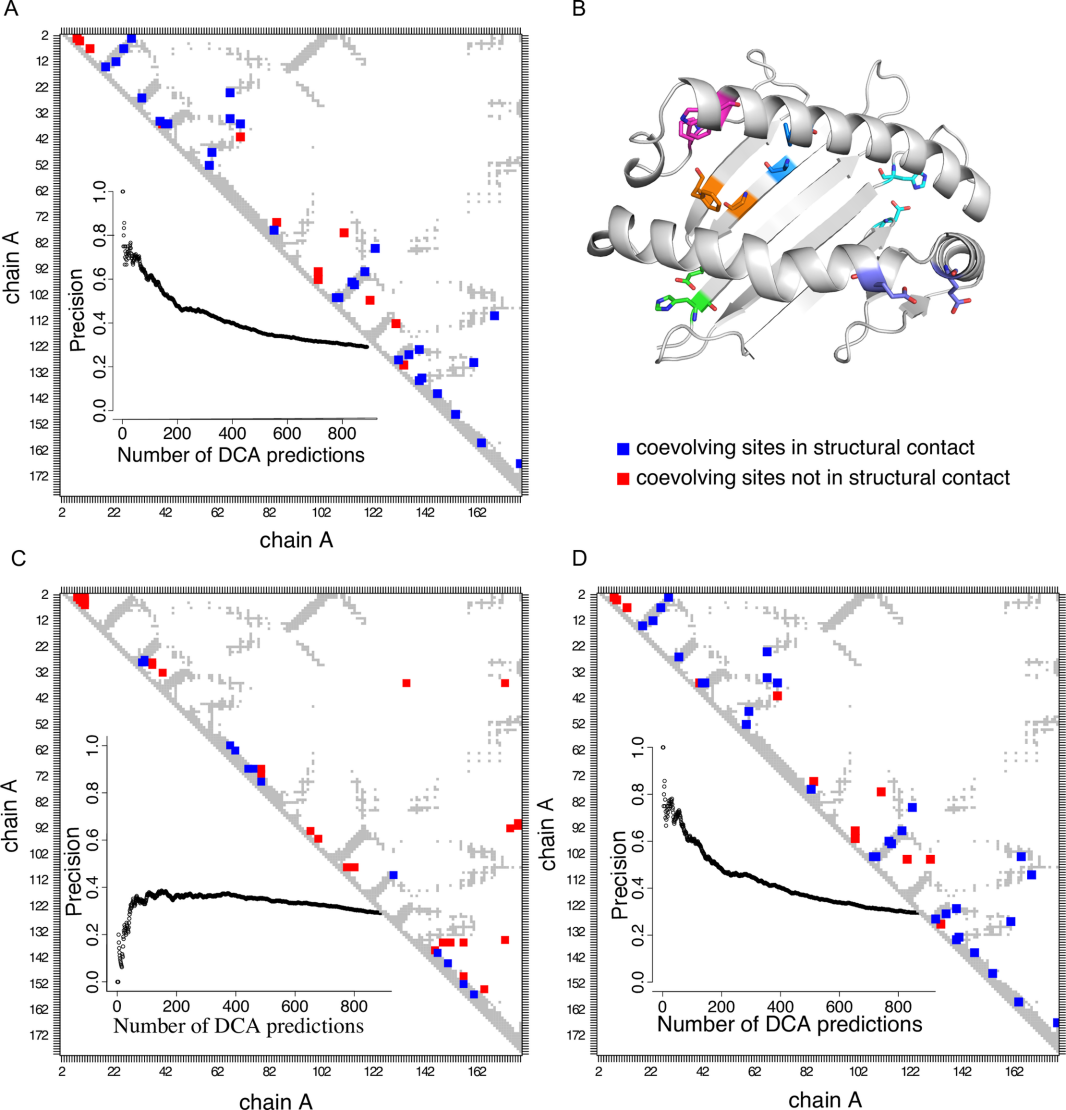
Inter- but not intra-species co-evolution accurately predicts structural contacts for MHC-I molecules. A. Contact map based on HLA-A02:01 structure (PDB: 2BNR, pairs of residues at distance < 8A are shown in grey) summarizing DCA predictions (top 44) with all vertebrate MHC-I sequences (see Materials and Methods). Blue squares represent structurally close pairs of sites predicted by DCA and red squares represent structurally distant pairs of sites predicted by DCA. The inset shows the precision (number of true positives divided by total number of predictions) for different numbers of DCA predictions (see Materials and Methods). B. Three-dimensional structure of HLA-A02:01 allele (PDB: 2BNR). The top six DCA predictions of co-evolving pairs of amino acids are displayed with different colours. C. Same data as in B, but restricting DCA predictions to human MHC-I sequences. D. Same data as in B, but restricting DCA predictions to non-human MHC-I vertebrate sequences.

To investigate co-evolutionary constraints among MHC-I residues we retrieved all MHC-I protein sequences from the PFAM v30 database (PF00129) [2]. This domain family covers the MHC-I domains alpha1 and alpha2 (179 amino acid) and is present in 445 organisms [2]. We excluded from the dataset 117 sequences from 14 bacterial and viral species (see Materials and Methods). We ended up with 40’739 sequences, including 20’256 sequences from human MHC-I alleles where the MHC-I polymorphism has been most studied (Fig 1). We then applied DCA on the whole PFAM alignment. Considering pairs of residues that are distant along the protein sequence (more than 4 residues apart), we observed a very strong enrichment of structural contacts among pairs of residues with high DCA scores (Fig 2A). For instance, among the top 44 DCA predictions (25% of MHC-I PFAM domain length), 31 correspond to pairs of residues less than 8Å apart in crystal structures (see Fig 2A and Materials and Methods). For illustration the top 6 DCA predictions (pairs 3–29, 93–119, 47–60, 26–33, 148–154 and 36–43, with residue numbering as in X-ray structures) are shown in Fig 2B. Similar results were obtained using plmDCA [25][26](see S1 Fig). Overall, our results indicate that high enrichment in structurally interacting pairs of residues can be obtained with DCA even for a domain family spanning a relatively low number of species (in our case only vertebrates).

### Co-evolutionary predictions and species predominance

To assess the contribution of the 20’256 human sequences to the co-evolution predictions, we led two additional experiments: one where the co-evolving scores based on DCA are evaluated using solely the 20’256 human sequences (Fig 2C) and another where the co-evolving scores are evaluated by excluding the human sequences from the analysis (Fig 2D). These experiments revealed that the top predictions of DCA applied to human sequences did not highlight pairs of residues close in protein structures (Fig 2C). Reversely, when excluding all human sequences DCA predictions of co-evolving sites remained almost unchanged and still pinpointed mainly pairs of sites in the structural proximity (Fig 2D). Similar results are obtained using a threshold of 5Å to define the contact map (S2 Fig). Moreover when removing the sequences from species with more than 500 MHC-I sequences (*Homo sapiens* (Human); *Macaca mulatta* (Rhesus macaque); *Macaca fascicularis* (Crab-eating macaque) (Cynomolgus monkey); *Acrocephalus schoenobaenus* (sedge warbler); *Parus major* (Great tit); *Macaca nemestrina* (Pig-tailed macaque); *Bos taurus* (Bovine); *Sus scrofa* (Pig), we still observed that many of the top co-evolving sites are in structural proximity (S3 Fig). Altogether these experiments suggest that the co-evolution signal captured by DCA reflects molecular constraints in the course of vertebrate evolution, and not constraints on polymorphic sites within one species. This is in line with the low weight on human sequences due to their high homology in DCA within the full alignment (see Fig 1). Nevertheless, the lack of structurally meaningful correlations when considering only human sequences suggest that little co-evolution is observed among them, although polymorphic sites are contacting each other in the MHC-I binding site, and therefore could potentially display some level of correlation reflecting structural constraints.

### Polymorphism and co-evolving sites

**Fig 3:**
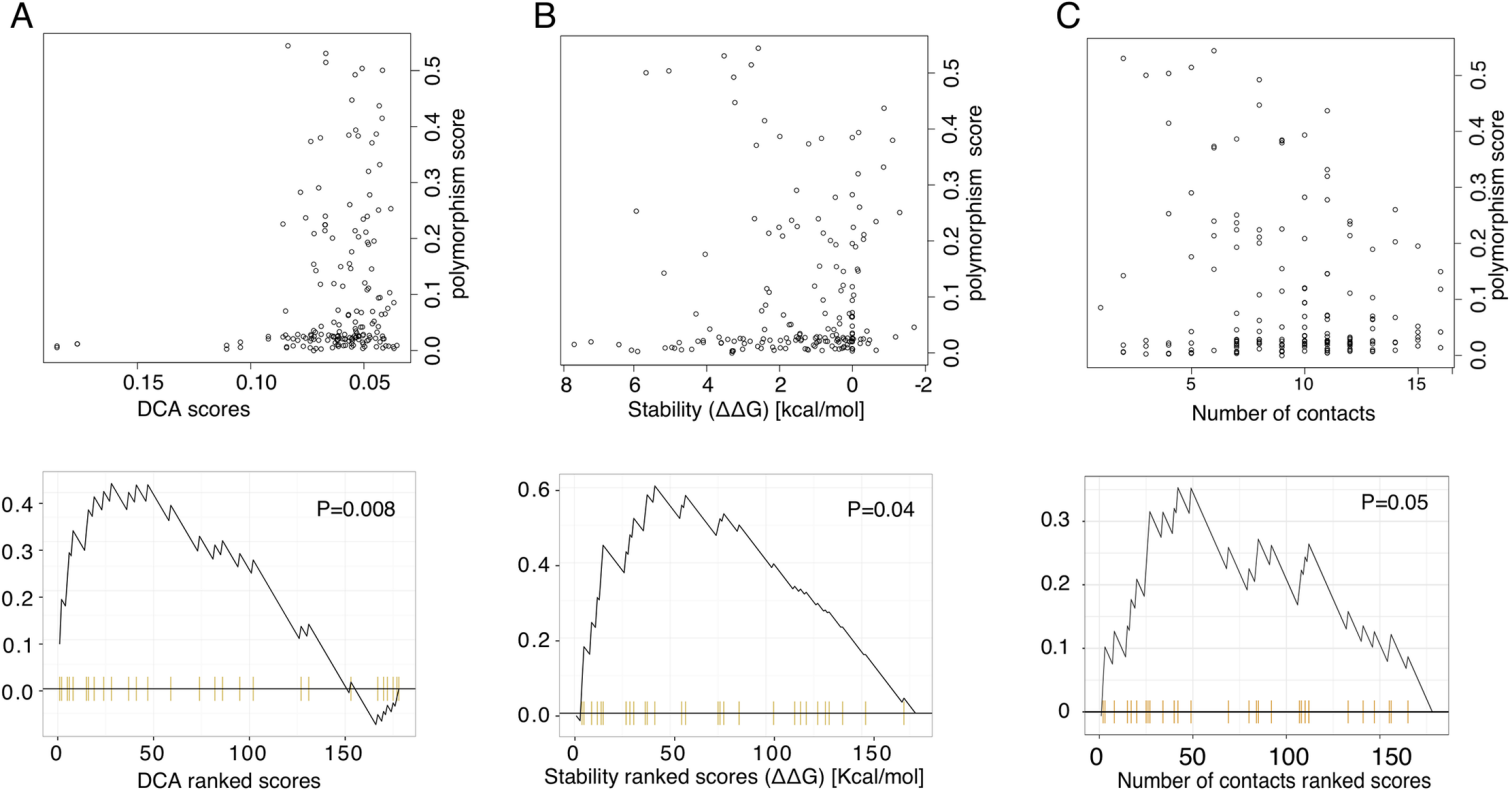
Polymorphic sites preferentially avoid co-evolving sites and sites involved in protein stability. **Top.** Plots of polymorphism scores versus: (A) DCA scores, (B) stability scores measured using FoldX (AlaScan function), (C) number of structural contacts. **Bottom.** Enrichment analysis of non-polymorphic sites with respect to (A) DCA scores, (B) stability scores and (C) number of structural contacts (x-axis shows the ranking of sites based on these values, sites with a polymorphism score lower than 0.01 are shown in yellow).

To further investigate the relationship between polymorphism and co-evolving sites, we measured conservation in human using information content (see Materials and Methods) to derive a polymorphism score for each site. A position with a minimal score is rarely mutated in human MHC-I alleles whereas a position with a high score is highly mutated. We then used Enrichment Analysis (see Materials and Methods) to determine the overlap (or absence thereof) between sites displaying strong co-evolutionary constraints across vertebrates as measured by DCA and polymorphic sites in human population. DCA scores were established for each site based on the highest DCA values with any other site more than 4 amino apart in the sequence, and sites where ranked based on these scores (x-axis in Fig 3A, lower panel) to compute the enrichment (or absence thereof) in polymorphic sites among sites with highest DCA scores. Using a threshold of 0.01 on the information content to define polymorphic sites, our analysis showed that pairs of sites with the highest DCA score mainly comprise sites that are non-polymorphic in human (Fig 3A, P = 0.008). This observation holds for threshold values of 0.02 and 0.03 (S4 Fig), or when defining polymorphic sites based on the most frequent MHC-I alleles in Caucasian population (see Materials and Methods and S5 Fig). Similar results would be obtain by taking a threshold of 0.1 on the DCA score and using Fisher’s exact test to probe the depletion of points in the upper left part of Fig 3A (P = 0.003). The advantage of the enrichment approach is that is does not require fixing a threshold on the DCA scores. We further note that the cloud of points for DCA values lower than 0.08 in Fig 3A was expected since the majority of DCA values obtained from any alignment are significantly bigger than zero. However, as observed in previous studies, only the top ranking pairs give meaningful information about structural contacts. This is the reason why we used enrichment analysis in this work, as opposed to correlation coefficient whose value would be dominated by the low DCA scores, which cannot be interpreted in terms of biologically meaningful co-evolutionary constraints.

### Polymorphism and stability

We then investigated the relationship between polymorphism and predicted importance for structural stability. Stability score of each site was evaluated using FOLD-X AlaScan software [10,27] using the X-ray structure of HLA-A02:01 in complex with a 9-mer ligand (PDB: 2BNR). Sites with different stability values were then used in the same enrichment analysis as before to compare with polymorphic sites. Here as well, we observed that polymorphic sites tend to be distinct from sites predicted to play a role in protein stability (Fig 3B, P = 0.04). This observation holds when considering other alleles and their corresponding pdb structures to evaluate stability score of each residue (Table 1). We further investigated the relationship between polymorphism and the number of structural contacts made by each residue (Materials and Methods). As expected from the stability analysis (Fig 3B), residues making many contacts tend on average to be enriched in non-polymorphic sites (Fig 3C), although the enrichment did not pass the 0.05 threshold for significance. In general, the fact that polymorphic sites that do not lead to dysfunctional proteins, such as those in MHC proteins, are less implicated in protein stability has been documented in many previous studies [28–32]. However, to our knowledge, our work is the first to perform such analysis specifically on MHC proteins.

**Table 1:** Enrichment of non-polymorphic sites with respect to stability evaluated in different structures. The p-values of enrichment analysis (column 3, also see Fig 3B) of non-polymorphic residues among sites contributing most to protein stability using different pdb structures (column 2) of MHC-I alleles (column 1) is shown below. For each pdb structure, we merged the peptide and the MHC-I allele on the same chain and ran AlaScan to measure the stability scores.

Allele	PDB	p-value
HLA-B51:01	1e27	0.01
HLA-C03:04	1efx	0.03
HLA-B44:02	1m6o	0.05
HLA-B44:03	1n2r	0.004
HLA-B27:05	1ogt	0.06
HLA-A11:01	1x7q	0.04
HLA-B35:08	2axf	0.02
HLA-B35:01	2cik	0.06
HLA-A01:01	3bo8	0.04
HLA-A02:03	3ox8	0.02
HLA-A02:07	3oxs	0.01
HLA-A03:01	3rl1	0.02
HLA-A24:02	3vxn	0.05
HLA-A68:01	4hwz	0.03
HLA-A68:02	4hx1	0.09
HLA-B18:01	4xxc	0.03
HLA-B27:04	5def	0.07
HLA-B07:02	5eo0	0.07
HLA-B57:01	5t6w	0.04

To assess whether co-evolving pairs of residues may simply reflect sites involved in protein stability, we investigated the relationship between DCA scores and either stability or number of contacts. We observed a very poor correlation between DCA scores and stability scores (S6A Fig) or number of contacts (S6B Fig). As expected, we observed a higher correlation between stability scores and number of contacts (S6C Fig). These results show that amino acid correlation patterns are not simply recapitulating the importance of residues for protein stability and could highlight distinct constraints that cannot be captured by stability predictions or number of structural contacts.

### Co-evolving constraints in the presence of peptide ligands

**Fig 4:**
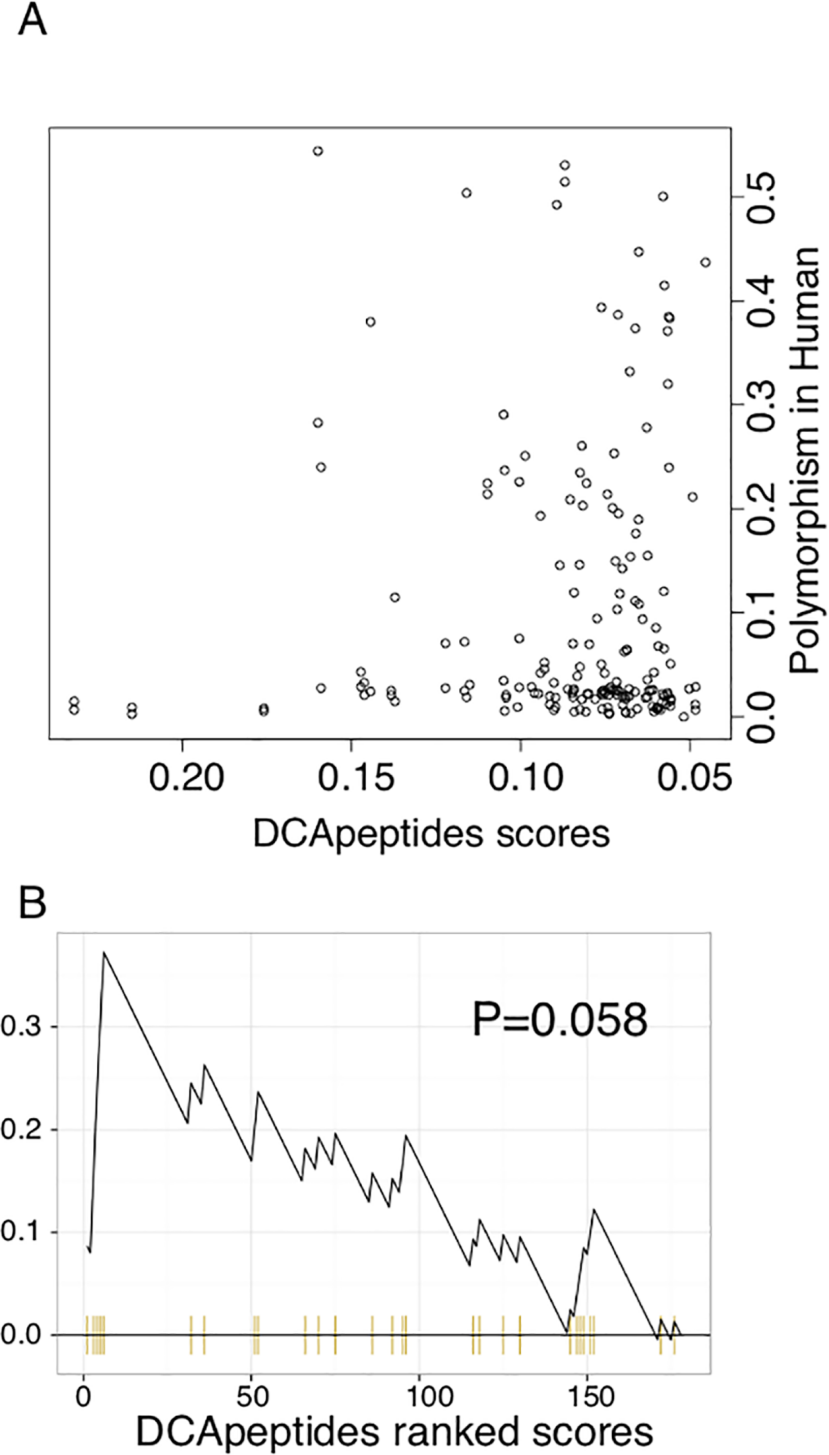
Co-evolution in the presence of peptides. **A.** Relationship between DCA scores and the polymorphism score when including predicted MHC-I ligands in the alignment and using the extended version of DCA (“DCApeptides”). The x-axis denotes the polymorphism score and the y-axis denotes DCApeptides co-evolution score (see Materials and Methods). **B.** Enrichment in non-polymorphic sites (threshold of 0.01) with respect to DCApeptides scores.

MHC-I molecules are known to interact with many peptides and the presence of a peptide is required for MHC-I folding. To explore the effect of the presence of peptide ligands on DCA predictions, we built an expanded version of DCA, called DCApeptides, that can take as input several peptide ligands for each protein sequence. The set of peptides interacting with a given protein are used to compute the single and paired frequencies used in DCA, as described in Materials and Methods. Although major efforts have been invested in the field to experimentally characterize the MHC-I binding specificity repertoire in human and mice [33–36], the vast majority of MHC-I molecules do not have experimental ligands. To fill this gap, we selected 100’000 random 9-mer peptides from several organisms and evaluated the predicted binding affinity of MHC-I sequences to each of these peptides using NetMHCpan3.0 [37] (see Materials and Methods). For each MHC-I sequence we then selected the top 2% of the peptides, following the cut-off currently suggested by the authors of NetMHCpan [37]. These predicted ligands were included in the co-evolution calculations using the DCApeptides algorithm. Overall, results did not change much and we still observed the decoupling between co-evolving and polymorphic sites (Fig 4). However, it should be noted that these are predicted ligands and the signal captured by DCApeptides reflects at best what is implicitly modelled in the predictor and not necessarily the real inter-molecular constraints.

### DCAPeptides for inter-molecular contact predictions

**Fig 5:**
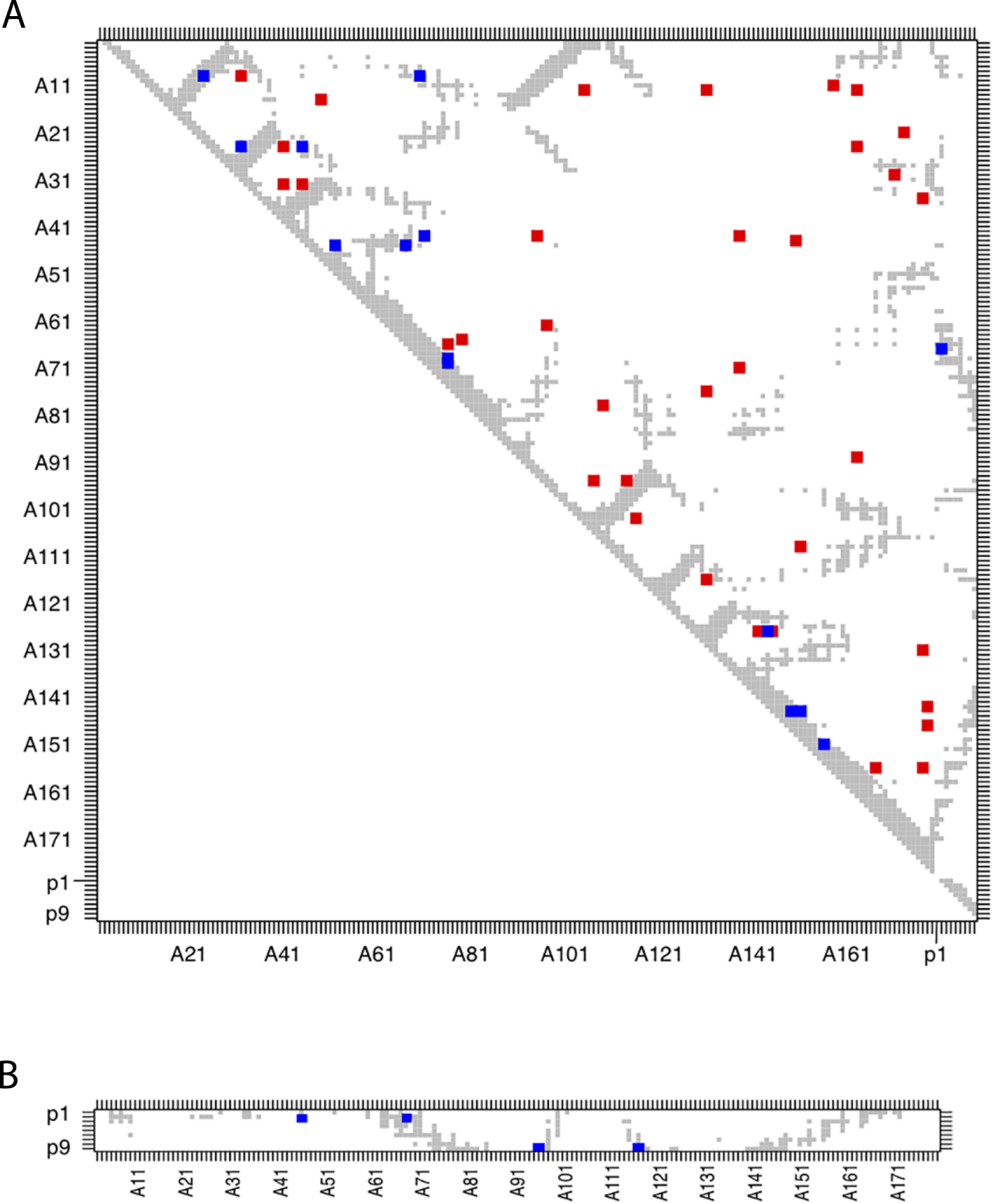
Co-evolution in human MHC-I sequences and their experimentally determined ligands. Contact map based on HLA-A02:01 structure (PDB: 2BNR, pairs of residues at distance < 8Å are shown in grey) summarizing DCApeptides predictions for the alignment of 156 human MHC-I molecules and their ligands. Chain A stands for the MHC-I sequence and chain P (P1-P9) for the ligands. Blue squares represent structurally close pairs of sites predicted by DCApeptides and red squares represent structurally distant pairs of sites predicted by DCApeptides. **A.** Co-evolution signal using the full alignment of MHC-I and their ligands (top 44 pairs). **B.** Inter-molecular co-evolution signal between MHC-I and their ligands (top 4 pairs).

To further explore the DCApeptides algorithm in the case of experimental ligands, we restricted the study to human MHC-I alleles having experimental ligands in IEDB [36] (see Materials and Methods). The number of such alleles is much smaller (156) and, as expected, we did not observe good structural contact predictions (Fig 5A). However, when restricting the analysis to inter-molecular pairs, we observed that the top 4 inter-molecular DCA pairs mapped accurately to existing structural contacts (Fig 5B). Moreover, these 4 pairs of sites involved residues P2 and P9 in the MHC-I ligands, which are known to be the main specificity determining residues (so-called anchor residues). Overall, our results indicate that DCApeptides predictions are stronger among MHC-I residues then between MHC-I residues and their ligands. However, DCA predictions among MHC-I residues do not pinpoint structural contacts (as in Fig 2C), while DCA predictions between MHC-I residues and their ligands revealed known interactions.

We further extended our benchmarking of the DCApeptides algorithm to the human PDZ protein domains, which are also known to interact with several ligands (in our dataset, these ligands came from a phage display experiment [38], see Materials and Methods). Here as well, we observed stronger correlation among the PDZ domain residues (S7A Fig). Some of the DCA predictions mapped to known structural contacts (15/27). More interestingly, when focusing only on correlations between PDZ residues and their ligands, we saw that DCApeptides could accurately predict some of the contacting pairs of residues. In particular, the top 2 predictions involved both position -2 in the PDZ ligands (S7B Fig), which is known to be the main specificity determining position for PDZ ligands [39]. Altogether, our results suggest that, when focusing on domains with available ligands from one species, intra-molecular DCApeptides predictions are not able to identify residues in structural proximity (likely because of the much lower number of sequences imposed by the constraint of having experimental ligands available), but inter-molecular DCApeptides predictions can accurately pinpoint structural contacts.

## Discussion

Co-evolution analyses have been widely used in biological studies, focusing mainly on co-evolution across species [14,40]. To our knowledge, our work is the first co-evolution analysis of a protein family that displays at the same time high variability between species and high polymorphism within species. As MHC-I polymorphism is known to be functionally important to entail different alleles with a wide range of binding specificities, our observation that polymorphic sites tend on average to show less co-evolutionary constraints may reflect the importance of preserving high mutability of these sites. It is also interesting to note that the de-coupling between polymorphic sites and co-evolving sites was even stronger than between polymorphic sites and sites involved in protein stability (Fig 3), suggesting that co-evolution constraints captured by DCA may be especially detrimental for polymorphic sites.

To predict co-evolving sites within MHC-I molecules, we used the DCA model introduced in [12,23], [22]. DCA demonstrated its statistical power on protein domains for which many homolog sequences are available (typically >10’000 sequences, ideally spanning both eukaryotes and prokaryotes) [22]. This study demonstrates that DCA predictions are highly enriched in structural contacts in MHC-I protein family, although the number of species is restricted to 445 (Fig 1). As in all DCA analyses, we focused here on sites that are distant in the sequence (i.e., more than 4 amino acids apart), which ensures that predictions of structural contacts are not simply resulting from sequence proximity. As such our work suggests that polymorphic sites tend to show less co-evolutionary constraints with sites distant in the primary sequence. Importantly, polymorphic sites have similar numbers of structural contacts with residues distant in the sequence (S8 Fig) as other residues, and therefore the observations made in this study could not simply be explained by the absence of such contacts.

The co-evolution signal detected in our analysis likely comes from the presence of divergent vertebrate species in the dataset, since very similar predictions were obtained by excluding the 20’256 human sequences in the datasets (Fig 2C and 2D), or by excluding species with more than 500 sequences in the dataset (S3 Fig). We anticipate that the fast evolutionary dynamic of MHC-I proteins may contribute to generating a stronger co-evolutionary pattern compared to other protein families, which could explain why we were able to detect it, although the MHC-I family is restricted to vertebrates.

DCA does not consider the actual phylogeny and takes only the alignment of sequences as input [14,18]. However, MHC-I evolution is difficult to characterize especially because it was subject to several duplication events along vertebrate evolution. Moreover the rate of evolution and the role of MHC-I in the immune system differ from one vertebrate species to another [41–43]) making it even more challenging to use available phylogenetic-dependent methods to predict co-evolving constrained sites since these models assume a homogeneous rate of substitutions across species evolution.

Ligands binding to MHC-I molecules play a role in MHC-I binding stability, which is why we included the ligands in stability predictions based on HLA-A02:01 structure. *In vivo*, MHC-I molecules are known to interact with tens of thousands of different peptides [33,44] and their specificity cannot be summarized with one single peptide. This is the reason why we extended the DCA framework to consider multiple ligands per protein in the alignment (Fig 4). Unfortunately, due to the scarcity of experimentally determined MHC-I ligands in most species except for human and mouse, the co-evolution analysis could not be carried out only with experimental ligands for all alleles included in our dataset. We therefore used for each allele 2’000 predicted ligands corresponding to the top 2% of a set of 100’000 peptides randomly selected from different proteomes [37]. As such, it is likely that the inter-molecular co-evolutionary signal observed in Fig 4 only captures the signal that is present in the NetMHCpan predictor, and may therefore not capture signals coming from more distant species that are not included in the training set of this algorithm. Nevertheless, he fact that the decoupling between polymorphic and co-evolving sites was observed both without and with ligands suggests that our results do not depend significantly on the presence of ligands in our analyses.

Our extension of the DCA algorithm to consider multiple ligands of the same protein further enabled us to analyse inter-molecular co-evolution for both MHC-I and PDZ proteins with experimentally determined ligands. Remarkably, in both cases, the inter-molecular predictions pinpointed structural contacts, whereas the intra-molecular predictions did not (for the majority of them, at least). Similar results were recently reported in a study of Antibody-antigen interactions [45], where maximum-entropy models such as DCA could help predicting affinity between antigens and antibodies, but not structural contacts within antibodies. We anticipate that our extension of DCA (available at: https://github.com/GfellerLab/DCApeptides) will contribute to future analyses of the differences between inter- and intra-molecular amino acid co-evolution patterns.

### Conclusion

MHC-I molecules have emerged recently in life history and are mainly restricted to vertebrate species. Despite the limited number of species that contain MHC-I genes, we observed that co-evolution constraints identified by statistical methods such as DCA accurately predicted several structural contacts. Moreover, we found that the co-evolution signal was dominated by inter-species amino acid changes and was not due to the variations between alleles within the same species (e.g., human). To our knowledge, this work is the first co-evolution analysis of a protein family that displays at the same time high variability between species and high polymorphism within species. Finally, our results suggest that MHC-I polymorphic sites, in addition to providing distinct binding specificities, preferentially avoid residues that show either high amino acid co-evolution patterns or play an important role in protein stability.

## Materials and methods

### MHC-I domain alignment

In this study, we analysed the PFAM domain family named *Histocompatibility antigen*, *domains alpha 1 and 2 of class I* with the identifier PF00129. In PFAM v30 the domain family was composed of a total of 40’856 protein sequences [2]. We removed 117 bacterial and viral sequences from the dataset and kept only vertebrate MHC-I for a total of 40’739 sequences. The human sequences constitute 49.7% of the family followed by the Rhesus macaque sequences that represent 4.9% of the family (Fig 1). We filtered highly gapped columns (>70%), and the final alignment corresponds to positions 2 to 179 in HLA-A02:01 allele (residue following the numbering in the crystal structures such as PDB:2BNR chain A).

We further collected the most frequent human alleles in the allele frequency database [46] for USA NMDP European Caucasian population (comprising a total of 1,242,890 individuals). 331 alleles had a frequency exceeding 0.00001 (97 HLA-A, 181 HLA-B and 55 HLA-C alleles).

### Direct coupling analysis

We used *Direct Coupling Analysis (DCA) model* [12] for the intra-molecular analysis of co-evolving sites within MHC-I domain family alignment. DCA uses as input the frequency *f*_*i*_*(A)* of amino acid A in column *i*, the frequency *f*_*j*_*(B)* of amino acid B in column *j*, and the joint frequency count *f*_*ij*_(*A*,*B*) for pairs of amino acid A and B in columns *i* and *j* within a protein alignment, for all pairs of position *i* and *j*. These frequencies are computed including reweighting of sequences with >80% sequence identity and pseudo counts equal to the effective number of sequences after reweighting, as described in [12]. The sum of weights displayed in Fig 1 for each clade corresponds to the sum of ‘*m*_*a*_’ values, where *m*_*a*_ represents to the weight of sequence *a* (see Morcos et al. [12]), and can be interpreted as the effective number of sequences in this clade. Julia’s version of PlmDCA [26][25] was run on the same alignment with default parameters. The algorithm starts by removing the duplicate sequences. Once these sequences were removed PlmDCA analysed 22954 sequences, with an effective number of sequences Meff equal to 173.44.

### Mapping DCA prediction on contact maps

As a reference structure for MHC-I domain, we used the structure of HLA-A02:01 in complex with a canonical 9-mer peptide (PDB: 2BNR; [47]). We consider that two sites are close in the structure if the distance between any of the heavy atoms is smaller or equal to 8Å, as suggested by the authors of the original DCA study [12], and built the contact map (grey dots in Fig 2). Similar contact maps were built using cut-off of 5Å in S2 Fig. To analyse the predictions of DCA with respect to structural contacts, we only considered pairs distant in the sequence (over 4 amino acids apart) and displayed in the contact maps of Fig 2 the top 44 predictions (25% of the MHC-I domain length). The performance plot in the insets were computed as follows:

1. Order the pairs of sites decreasingly based on DCA scores.
2. Compute the precision (i.e., true positives divided by the total number of DCA predictions) for numbers of predictions ranging from 1 to 900.

### DCA scores: From pairs to sites

DCA provides a score for every pair of sites. To reflect whether a site is under a co-evolutionary constraint we first ranked the scores in a decreasing order. We iteratively attributed individual score for each site as follow:

1. At the beginning none of the sites has an individual score (I). Given a site s, I_s_ = 0.
2. Remove the first pair p composed of sites s_1_ and s_2_ on the top of the sorted list where p_s1s2_ is the pair score.
3. Check if s_1_ has an individual score. If it has an individual score then go to step 4. If not, attribute an individual score to s_1_ such that I_s1_ = p_s1s2_.
4. Check if s_2_ has an individual score. If it has an individual score then go to step 5. If not, attribute an individual score to s_2_ such that I_s2_ = p_s1s2_.
5. Re-iterate from 2 to 4 until all pairs of site from the list are considered.

### Entropy and polymorphism

For human sequences in the PFAM alignment, we used one minus the Shannon entropy (i.e., $1+\sum_{A=1}^{20}f_{i}(A)log\{f_{i}(A)\}/log\{20\}$, where *f*_*i*_*(A)* stands for the frequency of amino acid *A* at position *i*) to measure the polymorphism score at each position [48]. This score has a minimal value of zero when all amino acid frequencies in a site are equal and a maximal score of one when only one perfectly conserved amino acid is found at a given position. We omitted the gaps from the entropy measure. The polymorphism analysis was also performed using only the most frequent human MHC-I sequences (331 alleles, see before). To this end the human alleles were aligned with MUSCLE [49] and amino-acid to compute the Shannon entropy were weighted by the allele frequency in the USA NMDP European Caucasian population.

### Stability score

To evaluate the structural stability impact of each residue, the AlaScan function of the FOLD-X software [10,27] was used to calculate the energy contribution of each residue. The structures were first repaired using RepairPDB function. The stability score of each site was measured using a reformatted pdb structure of 2BNR [47] where MHC-I residues from position 1 to 179 and the ligand were merged on chain A.

### Number of contacts

The number of contacts of each site was measured using the pdb structure 2BNR (HLA-A02:01 allele in chain A and the ligand). For a given site, the number of contacts is the number of residues that are maximum 5Å distant from this site in the crystallized structure.

### Enrichment analysis

Enrichment Analysis was used to investigate the relationship between polymorphic sites and sites displaying strong co-evolution constraints as estimated by DCA. A site was considered to be non-polymorphic in human alleles when its polymorphism score was lower than a threshold of 0.01 (see S4 Fig for results with other thresholds). To compute enrichment curves, sites were ranked based on their DCA score (x-axis in lower panels of Fig 3). Whenever a non-polymorphic site is encountered along the ranking (yellow bars), the enrichment curve goes up. Whenever a polymorphic sites is found the enrichment curve goes down. The same enrichment analysis was also applied to investigate the relationship between polymorphic sites involved in structural stability or sites displaying many contacts in the crystal structure of HLA-A02:01. For the enrichment analysis and p-value calculations, we use a weighted version of the Kolmogorov-Smirnov statistic with exponent measure equal to 1, as in all standard enrichment analyses [50].

### Extension of DCA to consider multiple ligands

To model the existence of multiple (predicted) ligands for each MHC-I protein, the amino acid frequencies *f*_*i*_ and *f*_*j*_ for all sites and joint frequencies *f*_*ij*_ for all pairs of sites (i.e. including both sites in the MHC and sites in the ligands) were computed. Following the nomenclature used in [12] the point frequency for position *i* in ligand is computed as:

$$f_{i}(A)=\frac{1}{M_{\mathrm{eff}}+\lambda }\left(\frac{\lambda }{q}+\sum_{a=1}^{M}\frac{1}{m^{a}}\sum_{n=1}^{N^{a}}\frac{1}{N^{a}}\delta_{A,L_{i,n}^{a}}\right)$$

where $L_{i,n}^{a}$ stands for the i^th^ amino acid in the n^th^ ligand of protein *a*, and *N*^*a*^ stands for the number of ligands of *a* and *M* stands for the number of MHC-I sequences. The joint frequency between position *i* in the protein and position *j* in the ligand is computed as:

$$f_{ij}(A,B)=\frac{1}{M_{\mathrm{eff}}+\lambda }\left(\frac{\lambda }{q^{2}}+\sum_{a=1}^{M}\frac{1}{m^{a}}\delta_{A,A_{i}^{a}}\sum_{n=1}^{N^{a}}\frac{1}{N^{a}}\delta_{B,L_{j,n}^{a}}\right)$$

Where $A_{i}^{a}$ stands for the i^th^ amino acid in protein *a*. Finally, the joint frequency between two ligand positions (*i* and *j*) is computed as:

$$f_{ij}(A,B)=\frac{1}{M_{\mathrm{eff}}+\lambda }\left(\frac{\lambda }{q^{2}}+\sum_{a=1}^{M}\frac{1}{m^{a}}\sum_{n=1}^{N^{a}}\frac{1}{N^{a}}\delta_{A,L_{i,n}^{a}}\delta_{B,L_{j,n}^{a}}\right)$$

The sequence reweighting (*m*^*a*^) corresponds to the number of sequences with more than 80% sequence identity to protein *a*, and was computed considering only the MHC-I sequence identity. This implies that each ligand has a weight equal to the weight of its protein (1ma) divided by the number of ligands of this protein (*N*^*a*^), in order to ensure proper normalization. The same pseudo-count λ=Meff=∑a=1M1ma was applied as in the standard DCA. In the case of 9-mer MHC-I ligands, this resulted in a total alignment of 178+9 = 187 positions, where the first 178 positions are characterized by a single amino acid at each position, while the last 9 positions are characterized by a distribution of amino acids for each MHC-I and each position in the ligands. All the rest of the DCA algorithm remains the same (inversion of the (187*20) x (187*20) covariance matrix and estimation of the Direct Information scores). The script to run these calculations can be accessed at: https://github.com/GfellerLab/DCApeptides.

### Prediction of MHC-I ligands

To explore the impact of MHC-I ligands on the enrichment analysis of Fig 3A, we attempted to run DCApeptides on the full alignment, including multiple peptide ligands for each MHC-I protein. Since the MHC-I ligand repertoire for the vast majority of MHC-I alleles in different species is still not experimentally available, we generated 100’000 random 9-mer peptides from 7 proteomes (*Anguilla anguilla*, *Bos taurus* (Bovine); *Gallus gallus*; *Homo sapiens* (Human); *Larimichthys crocea*; *Mus musculus* (mouse); *Tinamus Guttatus*) and predicted the binding affinity of MHC-I alleles to each of these peptides using NetMHCpan3.0 [37]. We then selected the top 2% predictions for each MHC-I allele in our alignment and computed the co-evolution patterns including these ligands based on DCApeptides (see above). Only MHC-I sequences without gaps at binding site positions used in NetMHCpan3.0 were considered (27,373 MHC-I sequences in total).

### Experimental MHC-I and PDZ ligands

Experimental MHC-I ligands were retrieved from IEDB [36]. In total 156 human MHC-I alleles had experimental ligands (annotated as “Positive-High”, “Positive-Intermediate”, “Positive-Low” or “Positive”). Only 9-mers were considered and these ligands were used with DCApeptides. X-ray structure of HLA-A02:01 (PDB:2BNR) in complex with a 9-mer peptide was used to compute the contact maps of Fig 5.

Experimental PDZ ligands were retrieved from a large phage display screen performed for 54 human PDZ domains [38]. All ligands were aligned at their C-terminus. The contact map in S7 Fig was computed based on the X-ray structure of DLG2 (PDB: 2HE2) [51].

## Supporting information

S1 Fig Predictions of plmDCA. **A.** Contact map based on HLA-A02:01 structure (PDB: 2BNR, pairs of residues at distance < 8Å are shown in grey) summarizing PlmDCA predictions (top 44) with all vertebrate MHC-I sequences (see Materials and Methods). Blue squares represent structurally close pairs of sites predicted by PlmDCA and red squares represent structurally distant pairs of sites predicted by PlmDCA. The inset shows the precision (number of true positives divided by total number of predictions) for different numbers of PlmDCA predictions (see Materials and Methods). **B.** Venn-diagram of the overlap between the top 44 positions that are identified by either DCA or PlmDCA.
(TIF)

S2 Fig Contact map with 5Å threshold. Contact map constructed as in Fig 2 but with 5Å threshold distance and summarizing DCA predictions. **A.** DCA prediction with all vertebrate MHC-I sequences. **B.** Only human sequences. **C.** All vertebrates MHC-I sequences except human sequences. Blue squares represent structurally close pairs of sites and red squares represent structurally distant pairs of sites among the top 44 DCA predictions. In A. B. and C., the insets show the precision over different thresholds for the number of DCA predictions (see Material and Methods).
(TIF)

S3 Fig Species predominance. Contact map summarizing DCA predictions (top 44) with vertebrate species that have less than 500 MHC-I sequences (see Fig 1) and using an 8Å distance. Blue squares represent structurally close pairs of sites and red squares represent structurally distant pairs of sites (see Materials and Methods section).
(TIF)

S4 Fig Enrichment analysis for different thresholds on the polymorphism score. Enrichment plots (exponent = 1) of non-polymorphic sites with respect to DCA score, stability estimates and the number of contacts using different thresholds to define polymorphic sites: 0.01 in A, 0.02 in B and 0.03 in C. Column 1: enrichment analysis using DCA scores measured using all vertebrate sequences. Column 2: enrichment analysis using stability score measured using HLA-A02:01 allele and its associated peptide. Column 3: enrichment analysis using number of contacts.
(TIF)

S5 Fig Polymorphic sites preferentially avoid sites involved in protein stability and co-evolving sites. Same analysis as in Fig 3, but using only the 331 most frequent human MHC-I alleles (frequency >0.00001 in Caucasian population) to define polymorphic sites (same threshold of 0.01 on the polymorphism score as in Fig 3).
(TIF)

S6 Fig Correlation between DCA scores, predicted stability and number of structural contacts. **A.** Correlation between DCA scores and stability predictions. **B.** Correlation between DCA scores and the number of contacts for each residue. **C.** Correlation between stability predictions and the number of contacts for each residue.
(TIF)

S7 Fig Co-evolution between PDZ domains and their ligands. Contact maps based on 2HE2 structure (pairs of residues at distance < 8Å are shown in grey) summarising DCApeptides predictions based on the alignment of 54 PDZ domains and their ligands. Chains A (PDZ domain) and P (ligands, positions -9 to 0) are both represented in the contact maps. Blue squares represent structurally close pairs of sites predicted by DCApeptides and red squares represent structurally distant pairs of sites predicted by DCApeptides. **A.** Co-evolution signal using the full alignment of human PDZ and their associated ligands (top 25 pairs). **B.** Inter-molecular co-evolution signal between PDZ sequences and their associated ligands (top 2 pairs).
(TIF)

S8 Fig Polymorphism and structural contact. Comparison between the number of structural contacts with residues distant in the sequence (more than 4 amino acids) for polymorphic and non-polymorphic sites.
(TIF)
