# Supplementary figures and images for: Polymorphic sites preferentially avoid co-evolving residues in MHC class I proteins
Source: PLoS Comput Biol. 2018 May 21;14(5):e1006188. doi: 10.1371/journal.pcbi.1006188 (PMC5983860; doi:10.1371/journal.pcbi.1006188)

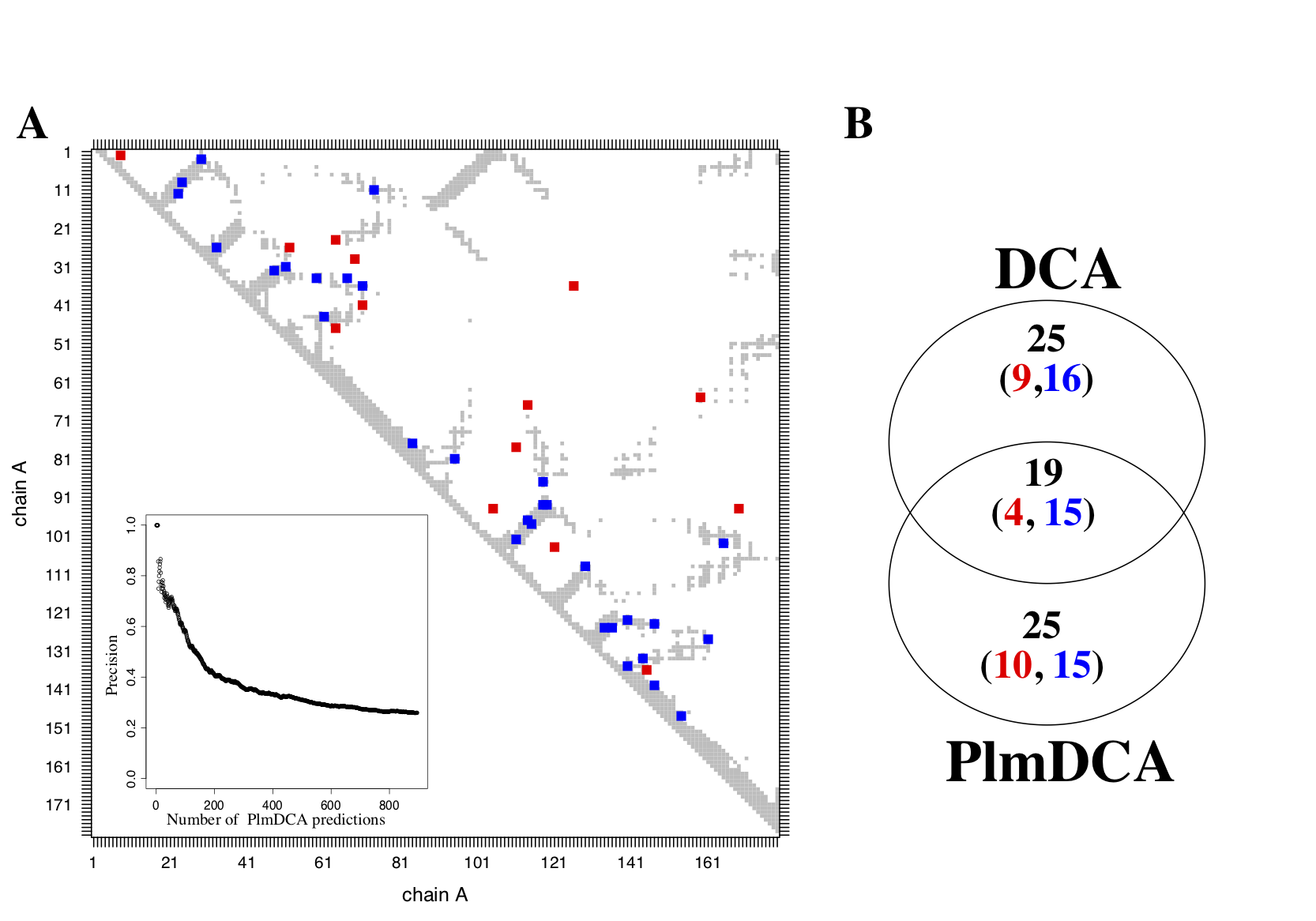

Supplement: S1 Fig — A. Contact map based on HLA-A02:01 structure (PDB: 2BNR, pairs of residues at distance < 8Å are shown in grey) summarizing PlmDCA predictions (top 44) with all vertebrate MHC-I sequences (see Materials and Methods). Blue squares represent structurally close pairs of sites predicted by PlmDCA and red squares represent structurally distant pairs of sites predicted by PlmDCA. The inset shows the precision (number of true positives divided by total number of predictions) for different numbers of PlmDCA predictions (see Materials and Methods). B. Venn-diagram of the overlap between the top 44 positions that are identified by either DCA or PlmDCA. (TIF) [file pcbi.1006188.s001.tif]

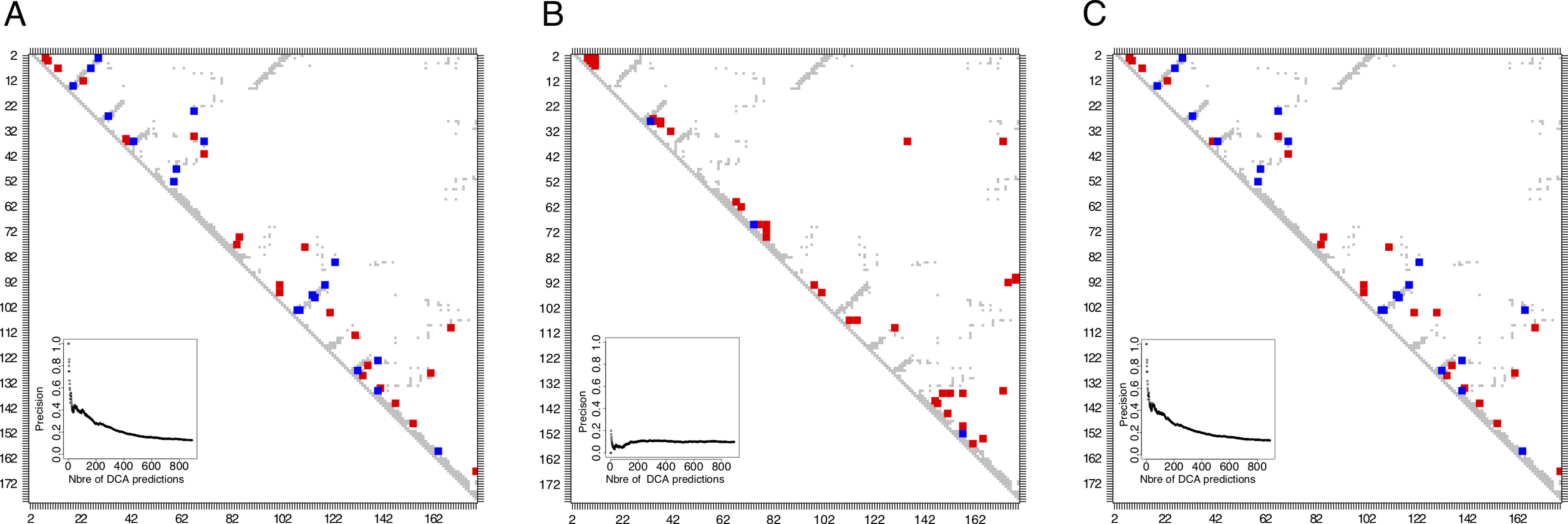

Supplement: S2 Fig — Contact map constructed as in Fig 2 but with 5Å threshold distance and summarizing DCA predictions. A. DCA prediction with all vertebrate MHC-I sequences. B. Only human sequences. C. All vertebrates MHC-I sequences except human sequences. Blue squares represent structurally close pairs of sites and red squares represent structurally distant pairs of sites among the top 44 DCA predictions. In A. B. and C., the insets show the precision over different thresholds for the number of DCA predictions (see Material and Methods). (TIF) [file pcbi.1006188.s002.tif]

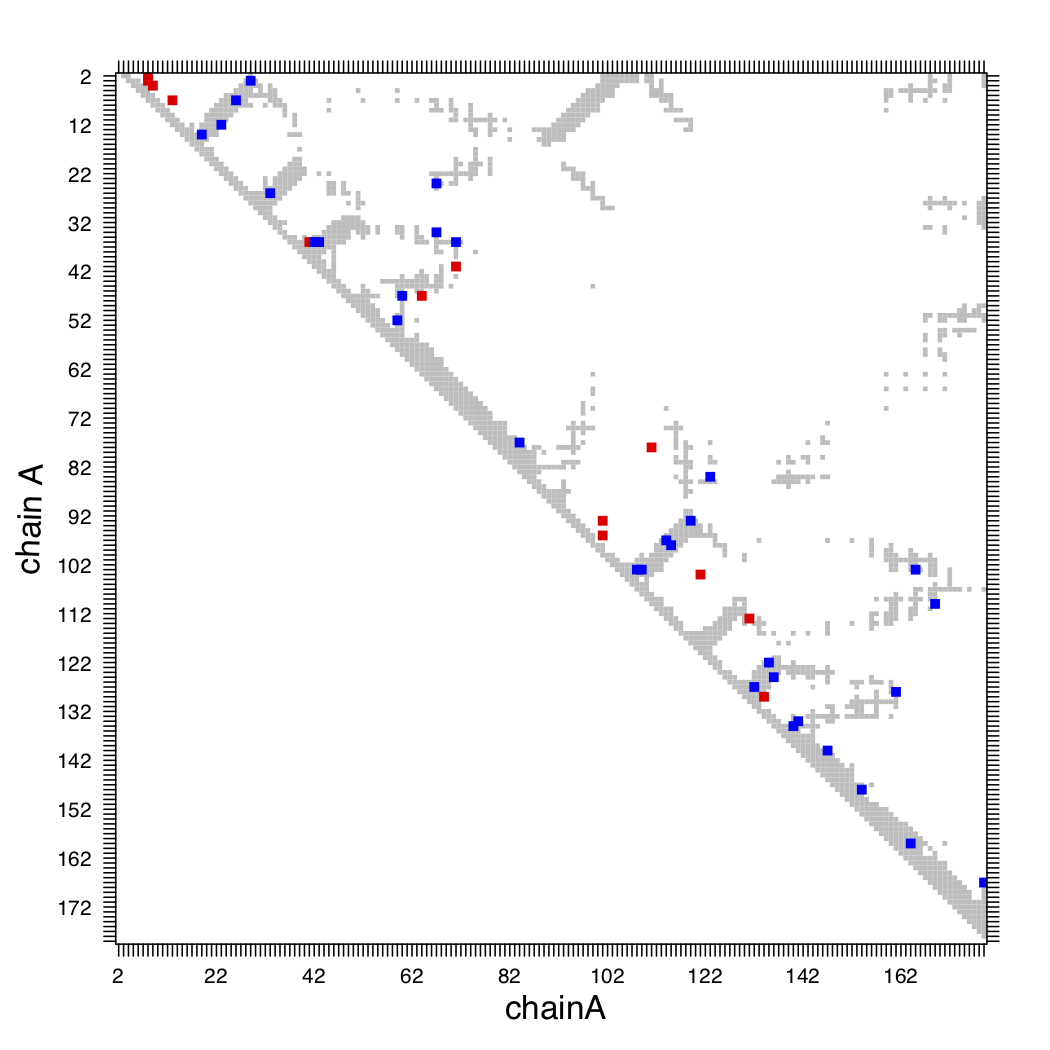

Supplement: S3 Fig — Contact map summarizing DCA predictions (top 44) with vertebrate species that have less than 500 MHC-I sequences (see Fig 1) and using an 8Å distance. Blue squares represent structurally close pairs of sites and red squares represent structurally distant pairs of sites (see Materials and Methods section). (TIF) [file pcbi.1006188.s003.tif]

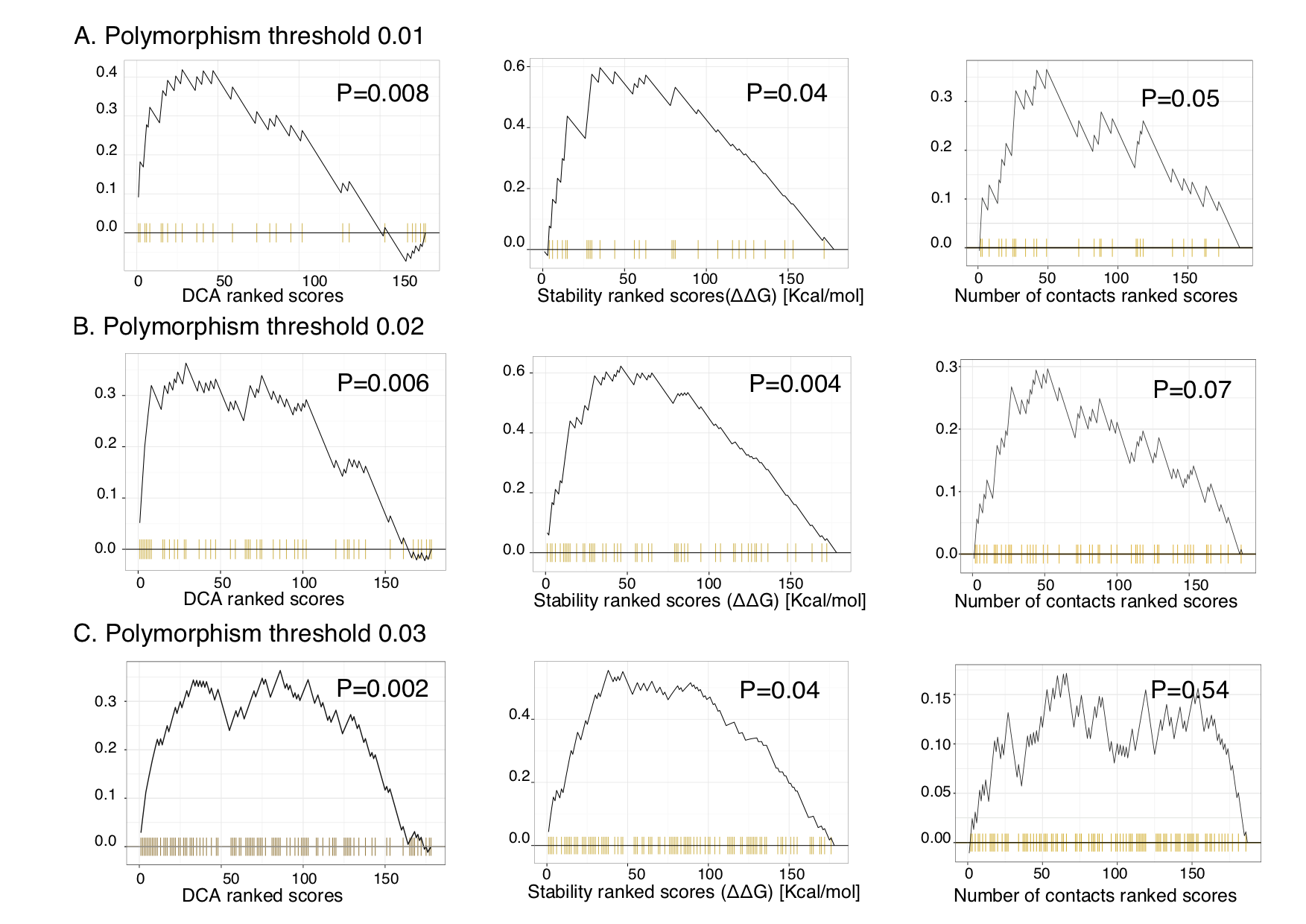

Supplement: S4 Fig — Enrichment plots (exponent = 1) of non-polymorphic sites with respect to DCA score, stability estimates and the number of contacts using different thresholds to define polymorphic sites: 0.01 in A, 0.02 in B and 0.03 in C. Column 1: enrichment analysis using DCA scores measured using all vertebrate sequences. Column 2: enrichment analysis using stability score measured using HLA-A02:01 allele and its associated peptide. Column 3: enrichment analysis using number of contacts. (TIF) [file pcbi.1006188.s004.tif]

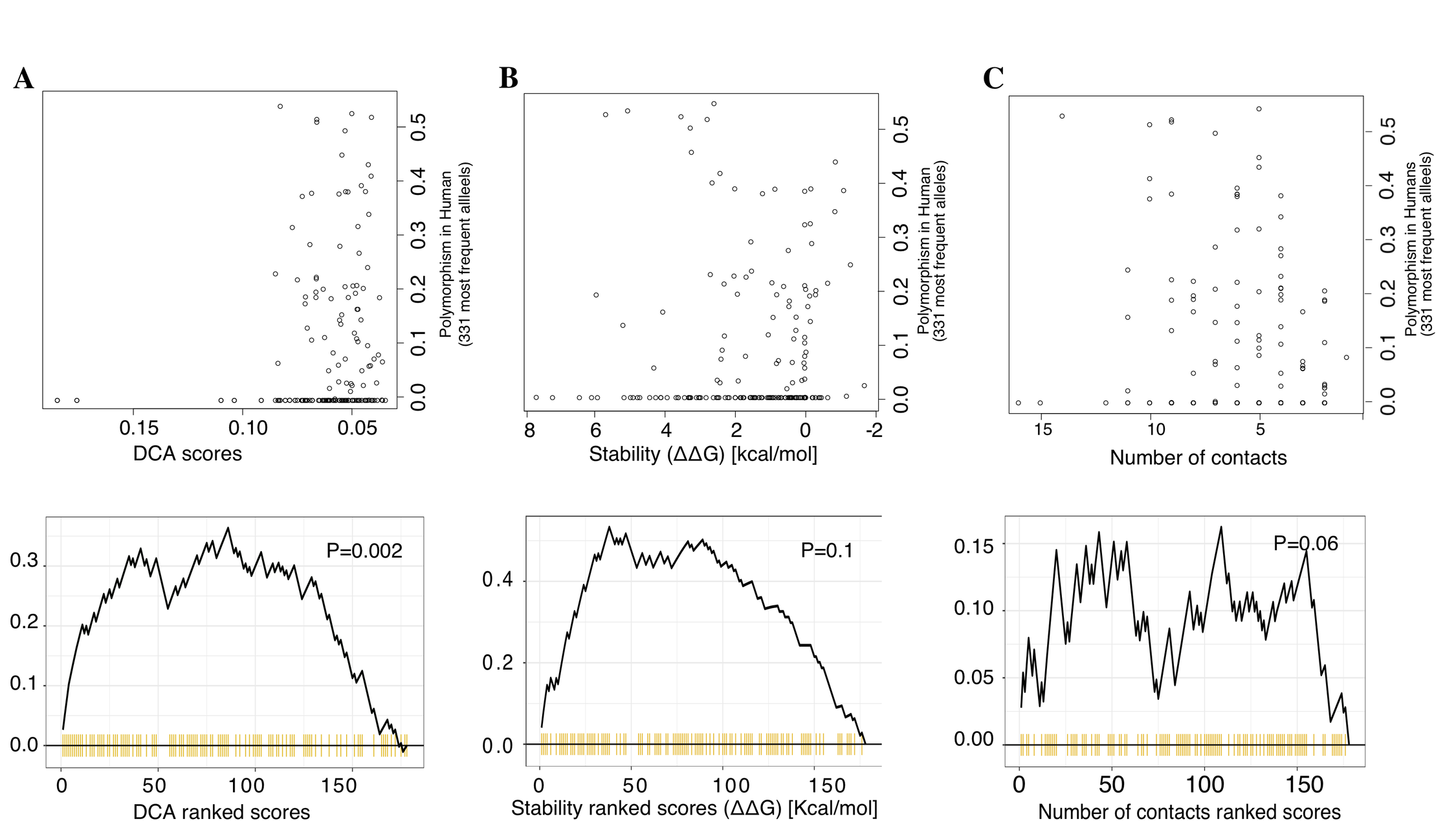

Supplement: S5 Fig — Same analysis as in Fig 3, but using only the 331 most frequent human MHC-I alleles (frequency >0.00001 in Caucasian population) to define polymorphic sites (same threshold of 0.01 on the polymorphism score as in Fig 3). (TIF) [file pcbi.1006188.s005.tif]

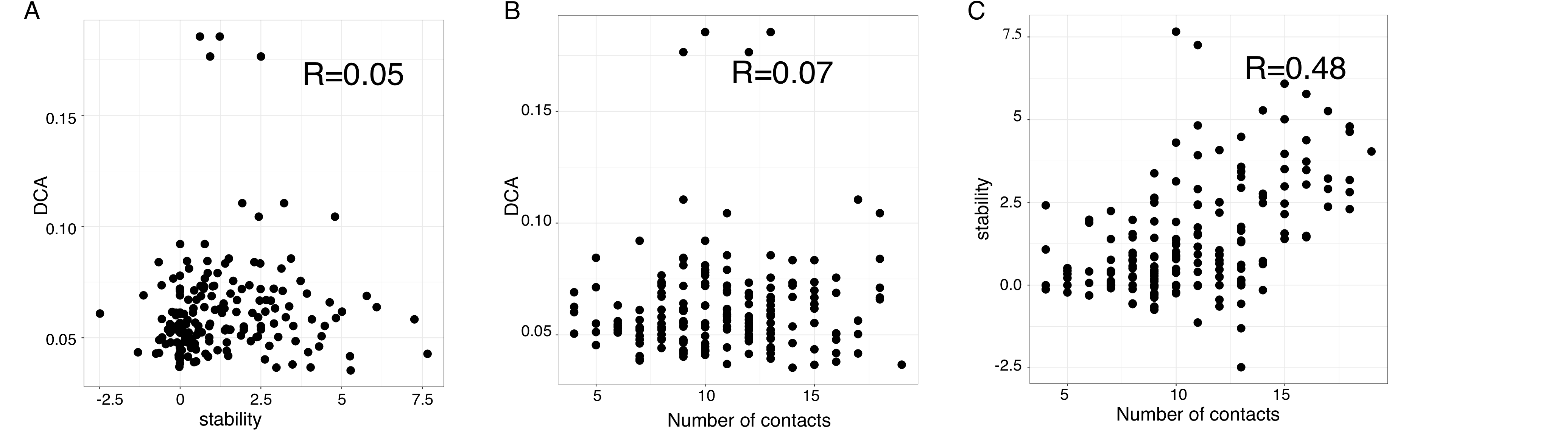

Supplement: S6 Fig — A. Correlation between DCA scores and stability predictions. B. Correlation between DCA scores and the number of contacts for each residue. C. Correlation between stability predictions and the number of contacts for each residue. (TIF) [file pcbi.1006188.s006.tif]

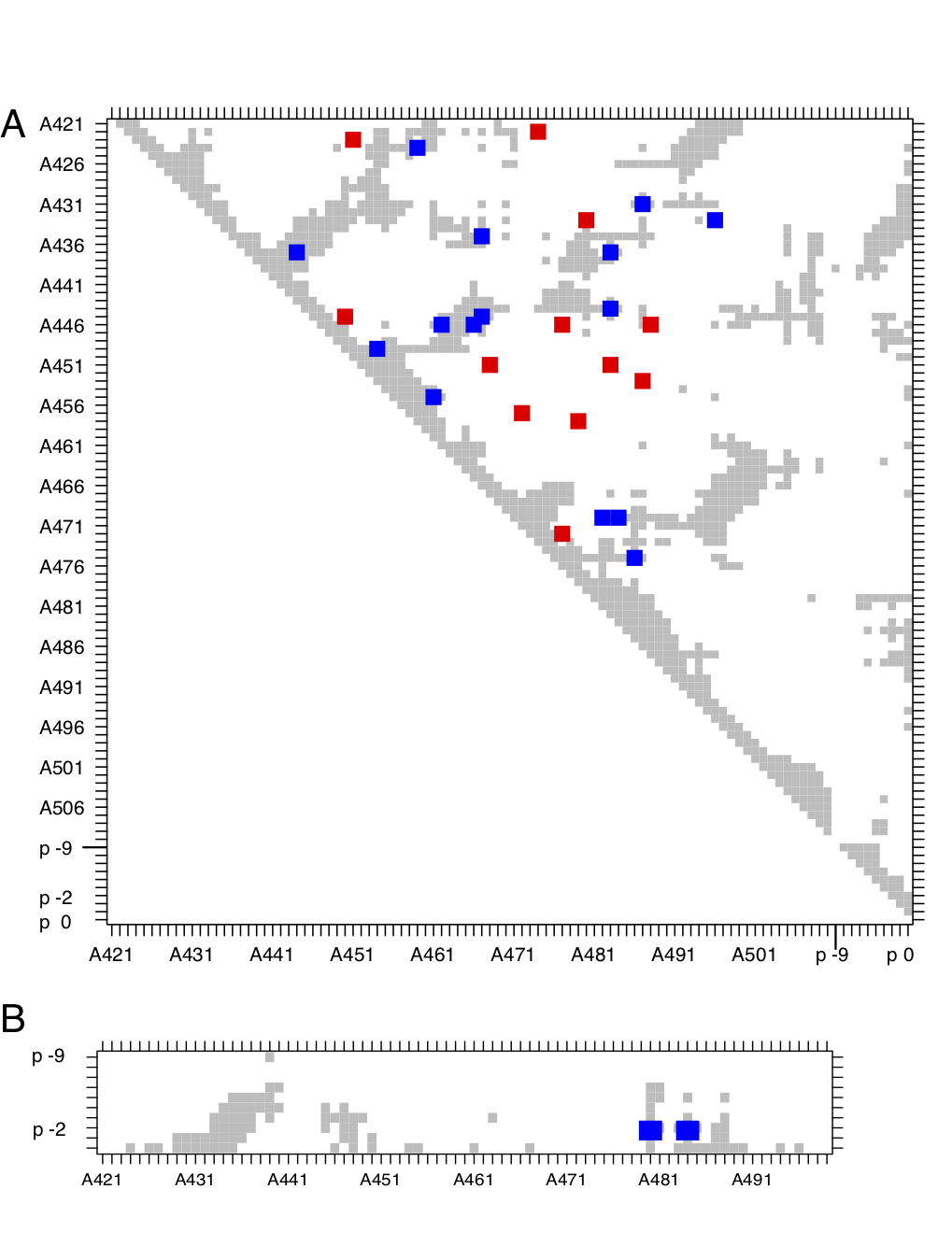

Supplement: S7 Fig — Contact maps based on 2HE2 structure (pairs of residues at distance < 8Å are shown in grey) summarising DCApeptides predictions based on the alignment of 54 PDZ domains and their ligands. Chains A (PDZ domain) and P (ligands, positions -9 to 0) are both represented in the contact maps. Blue squares represent structurally close pairs of sites predicted by DCApeptides and red squares represent structurally distant pairs of sites predicted by DCApeptides. A. Co-evolution signal using the full alignment of human PDZ and their associated ligands (top 25 pairs). B. Inter-molecular co-evolution signal between PDZ sequences and their associated ligands (top 2 pairs). (TIF) [file pcbi.1006188.s007.tif]

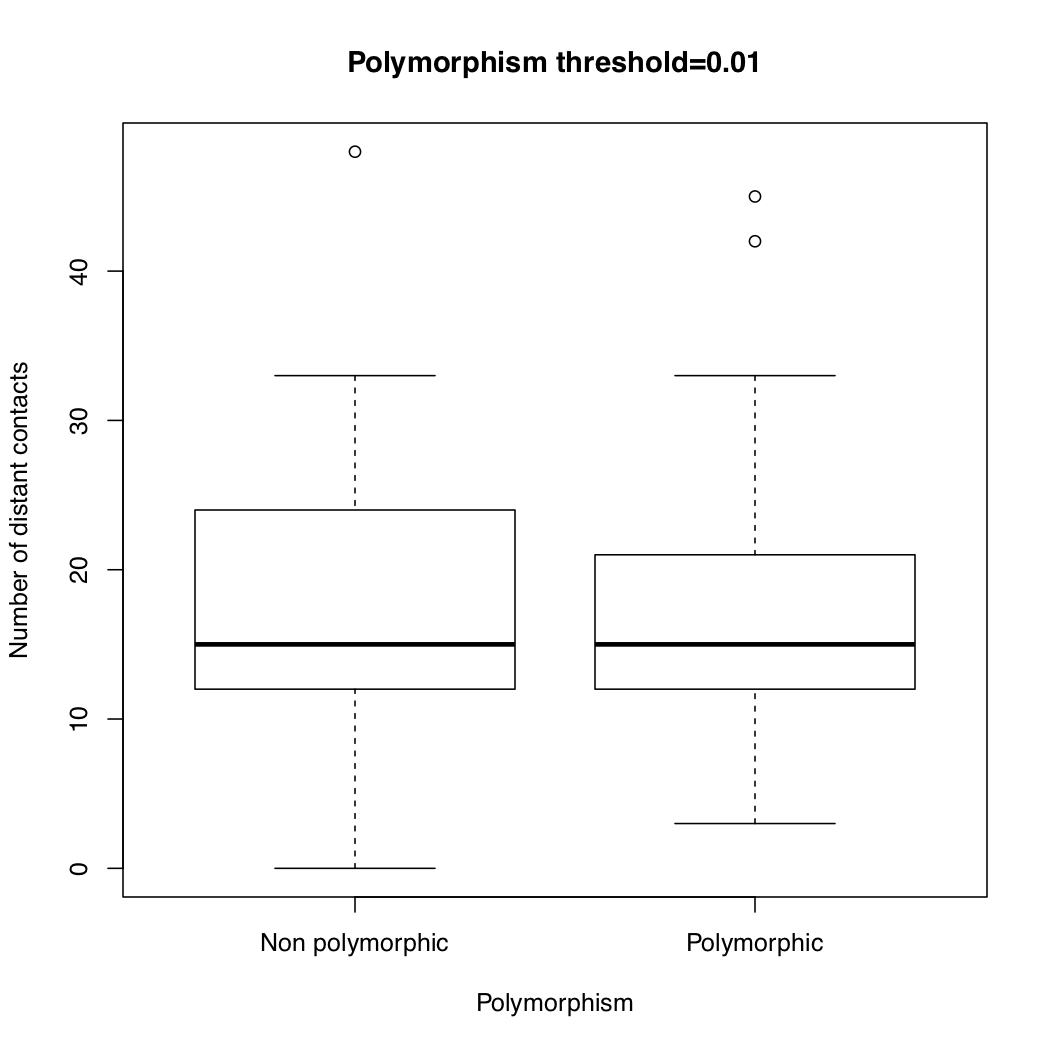

Supplement: S8 Fig — Comparison between the number of structural contacts with residues distant in the sequence (more than 4 amino acids) for polymorphic and non-polymorphic sites. (TIF) [file pcbi.1006188.s008.tif]
